# Genome Studies in *Amaranthus cruentus* L. and *A. hypochondriacus* L. Based on Repeatomic and Cytogenetic Data

**DOI:** 10.3390/ijms252413575

**Published:** 2024-12-18

**Authors:** Alexandra V. Amosova, Olga Yu. Yurkevich, Alexey R. Semenov, Tatiana E. Samatadze, Diana V. Sokolova, Anna M. Artemyeva, Svyatoslav A. Zoshchuk, Olga V. Muravenko

**Affiliations:** 1 Engelhardt Institute of Molecular Biology of Russian Academy of Sciences, 119991 Moscow, Russia; 2 Federal Research Center N.I. Vavilov All-Russian Institute of Plant Genetic Resources (VIR), 190000 St. Petersburg, Russia

**Keywords:** *Amaranthus* L., genome, satellite DNAs, FISH, molecular cytogenetic markers, karyotype structure, chromosome variability

## Abstract

*Amaranthus cruentus* L. and *Amaranthus hypochondriacus* L. are valuable and promising food crops for multi-purpose use that are distributed worldwide in temperate, subtropical, and tropical zones. However, their karyotypes and genomic relationships still remain insufficiently studied. For the first time, a comparative repeatome analysis of *A. cruentus* and *A. hypochondriacus* was performed based on the available NGS data; bioinformatic analyses using RepeatExplorer/TAREAN pipelines; and chromosome FISH mapping of 45S rDNA, 5S rDNA, and the most abundant satellite DNAs. In the repeatomes of these species, interspecific variations in the amount of Ty3/Gypsy and Ty1/Copia retroelements, DNA transposons, ribosomal, and satellite DNA were detected. In the repeatomes of both species, shared satDNAs with high sequence similarity were identified. The chromosome distribution patterns of four effective molecular markers, 45S rDNA, 5S rDNA, AmC4, and AmC9, allowed us to identify all chromosome pairs in the species karyotypes, construct unique karyograms of *A. cruentus* and *A. hypochondriacus*, and confirm the close relationship between their genomes. These results are important for comparative karyotypic studies within the genus *Amaranthus*. Our findings demonstrated that cytogenomic analyses might provide important data on genomic relationships within *Amaranthus* and increase knowledge on genome organization in these valuable crops.

## 1. Introduction

*Amaranthus* L. is a cosmopolitan genus of annual or short-lived perennial herbaceous plants. According to different reports, this genus includes from 60 to 100 species, which are distributed in the temperate, subtropical, and tropical zones [1]. *Amaranthus* species demonstrate high resistance to abiotic and biotic factors (e.g., diseases, drought, heat, salinity, etc.), and they could be valuable, promising donors of useful genes for developing new productive and resistant varieties of amaranths and other crops [2,3,4,5].

The *Amaranthus* species are recognized worldwide as an excellent source of biologically active substances, dietary fiber, and minerals, as well as easily digestible protein (up to 17%) [6,7,8]. Amaranth leaves and grains are superior in their nutritional properties and a number of important microelements, vitamins, and essential amino acids to most known and intensively used crops [8,9]. Amaranth seed oil contains tocopherol, tocotrienol, phytosterols, unsaturated fatty acids, squalene, aliphatic alcohols, terpene alcohols, isoprenoid compounds, polyphenols, and carotenoids, which have many health benefits [10]. In addition, amaranths are utilized as an effective feed crop for poultry (grain), as well as cattle and pigs (grazing, green feeding, and silage), due to the presence of beneficial compounds in their leaves and seeds [11].

Many amaranths are highly valued as medicinal plants. Seed oil is a powerful antioxidant and immune activator that can be used for the prevention and treatment of many viral and inflammatory diseases, metabolic disorders, diseases of the digestive system, and burns [8,12,13,14,15]. In addition, several *Amaranthus* species (e.g., *A. hypochondricus*, *A. cruentus*, *A. caudatus,* and *A. tricolor* L.) are used as ornamental plants due to their bright colors and unusual plant shapes [1].

The taxonomy history of the genus *Amaranthus* is rather complicated due to a wide range of intra- and interspecific phenotypic variability and the plasticity of its representatives, which could be related to the presence of various hybrids, possible events of introgressive hybridization, as well as the wide geographic range of amaranths [16,17,18,19,20]. In taxonomic reports, the genus *Amaranthus* is divided into three subgenera: *Acnida*, *Amaranthus*, and *Albersia* [19,21,22]. The most economically important subgenus, *Amaranthus* proper, involves several valuable species, including multi-purpose *A. hypochondriacus* L., *A. cruentus* L., and *A. caudatus* L., domesticated as grain crops (classified as pseudo-cereals); leafy vegetables; and medicinal, fodder, and ornamental plants in Central and South America, Africa, and Eurasia [1,23,24,25].

Currently, the genomes of amaranths are actively being investigated by various molecular approaches including the use of genetic markers: random amplified polymorphic DNA (RAPD) [26], simple-sequence repeats (SSRs) [27], bacterial artificial chromosome (BAC) libraries [28], single-nucleotide polymorphisms (SNPs), and genetic linkage maps [29,30]. For economically valuable amaranths, e.g., *A. cruentus*, *A. hypochondriacus* and *A. tricolor* L., a genome-wide assembly was carried out; genome sizes were estimated and transcriptomes were studied in order to investigate the origin and evolution of certain gene families and to identify biosynthetic gene clusters important for biotechnology [5,31,32,33,34,35,36]. According to different molecular phylogenetic evaluations, *A. hybridus* L., *A. quitensis* Kunth, and *A. powellii* S. Wats. are considered possible basal ancestral species [16,30,37,38]. Analyses of restriction site variations in nuclear and cytoplasmic DNA have shown that *A. caudatus* and *A. cruentus* are more closely related to each other and to their supposed progenitors than to *A. hypochondriacus* [39]. At the same time, a phylogenetic evaluation of genetic diversity using competitive allele SNP markers, conducted during the collection of cultivated and wild species, demonstrated that *A. cruentus* and *A. hypochondriacus* are related. However, both species are distant from *A. caudatus*, which, in turn, is closely clustered with a weedy relative, *A. quitensis* [30]. A genome assembly in *A. hypochondriacus*, *A. cruentus*, and *A. tricolor* indicated a whole-genome duplication event shared in the last common ancestor of the subfamily Amaranthoideae, and also confirmed the close relationships between these species [35,36,37].

The study of basic chromosome numbers in the karyotypes of amaranths did not clarify their phylogenetic relationships. Three basic chromosomal numbers were revealed within the genus *Amaranthus*: n = 14, 16, and 17 [1,40,41,42,43]. Most species are diploid except for one tetraploid species, *A. dubius* Mart ex. Thell. (2n = 4x = 64) [44]. Moreover, intraspecific variability in chromosome numbers were revealed in several species, including *A. caudatus*, *A. cruentus*, and *A. hybridus* [42,45]. Molecular cytogenetic studies using FISH (fluorescence in situ hybridization) were conducted on several *Amaranthus* species [45,46]. Depending on the species, 1–3 pairs of chromosomes bearing 45S rDNA signals and 1–6 chromosome pairs with 5S rDNA loci were revealed [45,46]. Intra- and interspecific variability in the number and chromosome distribution patterns of rDNA sites and constitutive heterochromatin (using the CMA_3_/DAPI banding technique) was observed in several amaranth species [41,45,46]. At the same time, the small sizes of chromosomes (0.8–3.5 μm) [40,41] make comprehensive molecular cytogenetic analyses difficult, and karyotypes of amaranths, including economically valuable species, still remain insufficiently studied. To obtain more detailed information about the structure of amaranth karyotypes, modern cytogenomic approaches are required.

Repetitive DNA makes up a major and fast-evolving portion of eukaryotic genomes that can drive genome evolution and regulate gene expression [47,48]. The RepeatExplorer pipeline/TAREAN pipelines are effective computational tools for the genome-wide characterization of repetitive DNA sequences from NGS (next-generation sequence) data and the identification of different satellite DNA families (satDNAs) from unassembled reads [49,50,51]. Based on these identified satDNAs, oligonucleotide FISH probes can be developed, which is important for molecular cytogenetic studies [52]. This approach can help identify new chromosome markers necessary for the cytogenomic study of plants (especially those with small-sized chromosomes) [53,54].

In the present study, for the first time, we performed a comparative characterization of the repeatomes of two economically valuable *Amaranthus* species, *A. cruentus* and *A. hypochondriacus*, which included the bioinformatic analysis of available high-throughput DNA sequencing data using RepeatExplorer/TAREAN pipelines and the Basic Local Alignment Search Tool (BLAST). Moreover, in these species, FISH chromosome mapping of the identified satDNAs, together with classical molecular cytogenetic markers (45S rDNA and 5S rDNA), was conducted to assess chromosomal variability and evaluate the genomic relationships between these species.

## 2. Results

### 2.1. Comparative Analyses of the Repetitive DNA Sequences Identified in Genomes of the Studied Species

**Figure 1:**
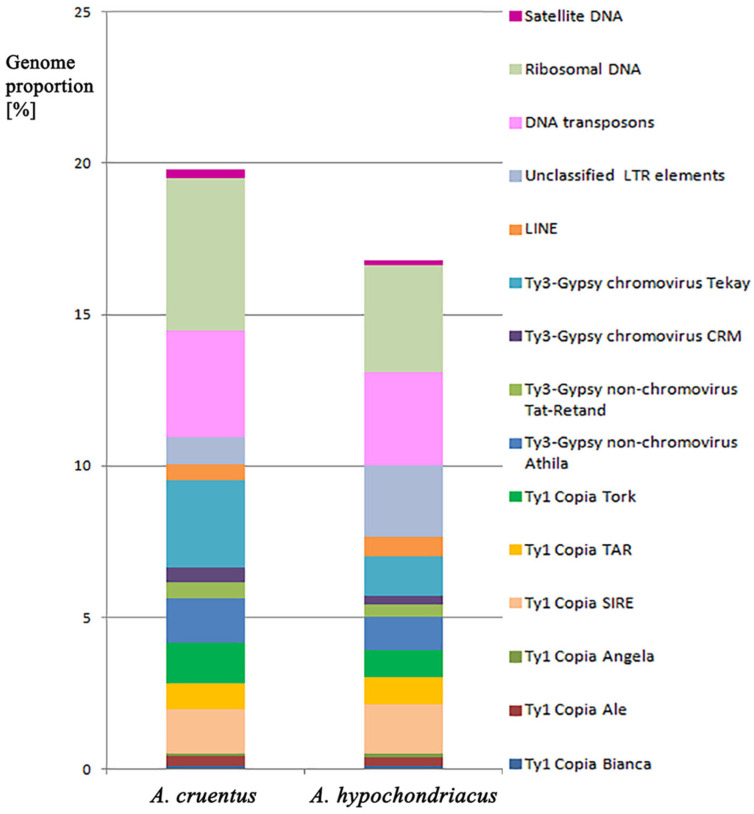
Types and genome proportions of the most abundant DNA repeats identified in the studied *Amaranthus species*. Each proportion was calculated using RepeatExplorer as the ratio of the number of reads specific to a particular repeat type to the sum of all reads used in the cluster analysis.

Analyses of the repeatomes of *A. cruentus* and *A. hypochondriacus* showed that mobile genetic elements made up the majority of their repetitive DNA (Figure 1, Table 1). In both *A. cruentus* and *A. hypochondriacus* genomes, retrotransposons (Class I) were rather abundant (11.06% and 10.07%, respectively). In *A. cruentus*, Ty3-Gypsy retroelements (5.37%) significantly exceeded Ty1-Copia retrotransposons, and in *A. hypochondriacus*, Ty1-Copia retroelements were more abundant than Ty3-Gypsy elements (3.03%). In the Ty1-Copia superfamily, SIRE (1.43% and 1.62%, respectively), Tork (1.35% and 0.93%, respectively) and TAR (0.86% for both species) were the most abundant retroelements. In the Ty3-Gypsy superfamily, chromovirus Tekay (2.90% and 1.3%, respectively) and non-chromovirus Athila (1.44% and 1.07%, respectively) were most abundant (Figure 1, Table 1).

In both species, DNA transposons (Class II) were found in lower amounts (3.52% and 3.07%, respectively) compared to retrotransposons, and within transposons, CACTA (0.82% and 2.24%, respectively) and MuDR_Mutator elements (1.46% and 0.77%, respectively) were dominated. In the genomes of both *A. cruentus* and *A. hypochondriacus*, large proportions of ribosomal DNA (5.06% and 3.54%, respectively) were revealed. At the same time, satellite DNA was not very abundant (0.27% and 0.16%, respectively) (Figure 1, Table 1).

Using TAREAN, 3–6 high-confident and 4–7 low-confident putative satDNAs were revealed in the genomes of the studied species (Table 1).

### 2.2. BLAST Similarity of the Identified SatDNAs

Among the identified satDNAs, the most abundant were AmC4, AmC9, AmC12, and AmC27 (*A. cruentus*), as well as AmH4, AmH9, AmH26, and AmH51 (*A. hypochondriacus*). According to TAREAN, they had genome proportions ranging from 0.23% to 1.5% (Table 2). BLAST (Basic Local Alignment Search Tool) revealed homologous satDNAs in the repeatomes of both *A. cruentus* (AmC9 and AmC27, 86% identity) and *A. hypochondriacus* (AmH9 and AmH51, 85% identity). Moreover, the most abundant repeats, AmC4, AmC9, and AmC12, were homologous (97–100% identity) to AmH51, AmH4, and AmH26, respectively. BLAST also determined the homology (88–95% identity) of AmC4, AmC9, and AmC12 repeats with different transcribed RNA sequences identified in the *A. tricolor* genome (Table 2). AmC12 covered 100% of the length of the mRNA (Sequence ID: XM_057683543.1); interspersed tandem AmC4 covered 38% of the length of the ncRNA (Sequence ID: XR_009039170.1); and AmC9 covered 9% of the length of the ncRNA (Sequence ID: XR_009042247.1) (Table 2, Supplementary Figures S1–S3, Supplementary Files S1–S3).

### 2.3. Chromosomal Structural Variations

**Figure 2:**
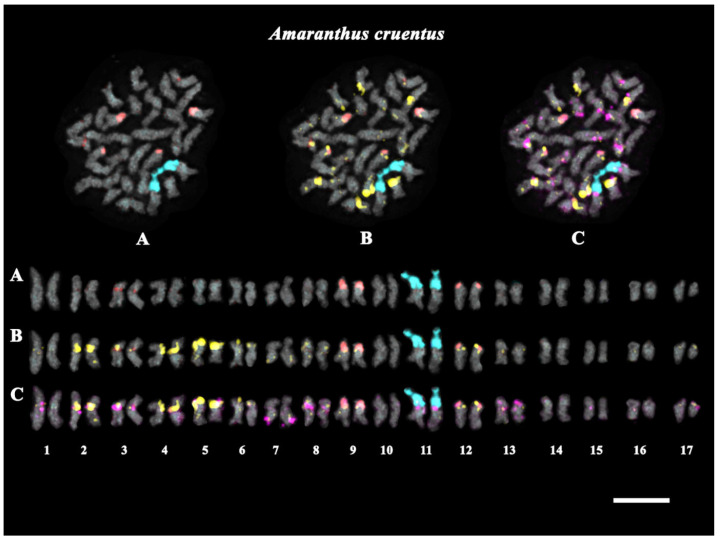
FISH-based localization of (**A**) 45S rDNA (aqua) and 5S rDNA (red); (**B**) 45S rDNA (aqua), 5S rDNA (red), and AmC4 (yellow); and (**C**) 45S rDNA (aqua), 5S rDNA (red), AmC4 (yellow), and AmC9 (purple) in the karyotype of *Amaranthus cruentus*. DAPI-staining—dark gray. Scale bar—5 μm.

**Figure 3:**
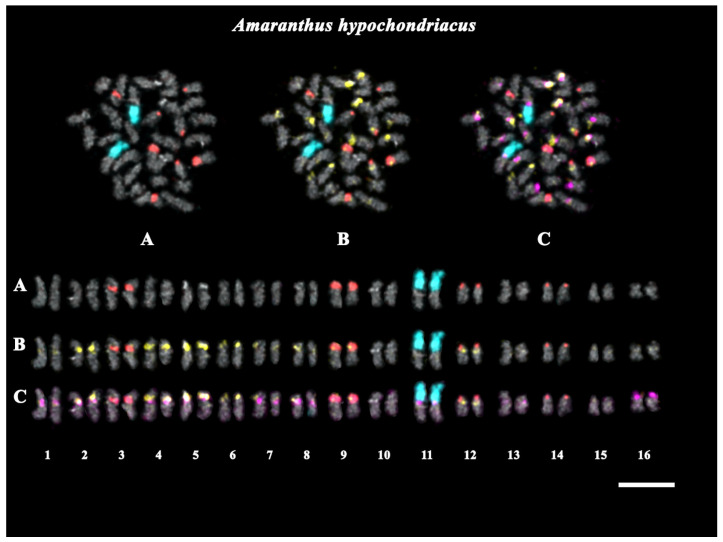
FISH-based localization of (**A**) 45S rDNA (aqua) and 5S rDNA (red); (**B**) 45S rDNA (aqua), 5S rDNA (red), and AmC4 (yellow); and (**C**) 45S rDNA (aqua), 5S rDNA (red), AmC4 (yellow), and AmC9 (purple) in the karyotype of *Amaranthus hypochondriacus*. DAPI-staining—dark gray. Scale bar—5 μm.

**Figure 4:**
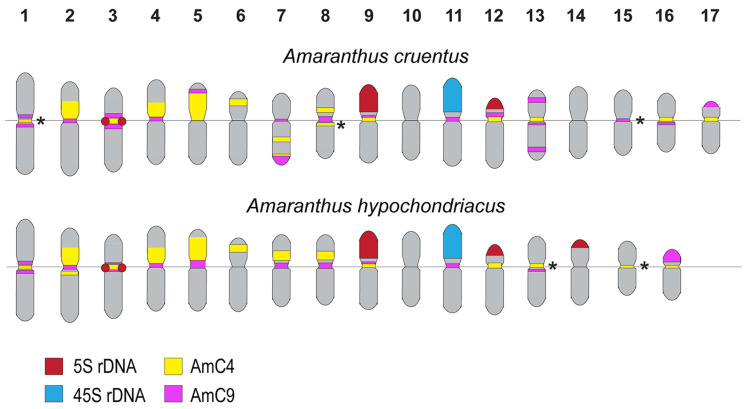
Ideograms demonstrating the positions of 45S rDNA (blue), 5S rDNA (red), AmC4 (yellow), and AmC9 (purple) sites in the chromosomes of *Amaranthus cruentus* and *Amaranthus hypochondriacus*. Asterisks indicate polymorphic sites.

In the present study, we analyzed the chromosomal organization of karyotypes of *A. cruentus* and *A. hypochondriacus* based on multicolor FISH procedures with 45S rDNA; 5S rDNA; and the oligonucleotide probes AmC4, AmC9, and AmC12, which were designed based on the four most abundant satDNAs repeats identified in the *A. cruentus* genome (Table 2). According to the FISH results, oligonucleotide probe AmC12 presented dispersed localization in the karyotypes of both studied species (Supplementary Figure S4). At the same time, we observed common features, as well as differences, in the patterns of the chromosomal distribution of 45S rDNA, 5S rDNA, AmC4, and AmC9. Based on the chromosome morphology and chromosome distribution patterns of these chromosome markers (45S rDNA, 5S rDNA, AmC4, and AmC9), homologous chromosome pairs were identified in the karyotypes of both species; and the species karyograms and idiograms, demonstrated the localization of the studied chromosome markers, were constructed (Figure 2, Figure 3 and Figure 4; Supplementary Figure S4).

The performed analyses showed that the studied *Amaranthus* specimens presented diploid karyotypes with 2n = 34 (*A. cruentus*) and 2n = 32 (*A. hypochondriacus*) chromosomes (Figure 2 and Figure 3). In *A. cruentus* and *A. hypochondriacus*, similar patterns of chromosome distribution for the 45S rDNA clusters were observed. Bright 45S rDNA signals were detected in the short arms of chromosome pair 11. In both species, major clusters of 5S rDNA were observed in chromosome pairs 3 (in the pericentromeric region), 9 (in the proximal regions of the short arms), and 12 (in the terminal regions of the short arms). In *A. hypochondriacus*, a major cluster of 5S rDNA was also revealed in the terminal regions of the short arms of chromosome pair 14 (Figure 2, Figure 3 and Figure 4).

In the karyotypes of both *A. cruentus* and *A. hypochondriacus*, co-localized clusters of AmC4 and AmC9 were revealed in the pericentromeric regions of chromosome pairs 1–4, 8, 9, and 13. Clusters of AmC4 were also detected in the pericentromeric regions of chromosome pairs 5, 12, and 16 and in the distal regions of the short arms of chromosome pair 6. Clusters of AmC9 were revealed in the pericentromeric region of chromosome pair 7 and in the secondary constriction region of chromosome pair 11 (Figure 2, Figure 3 and Figure 4).

Moreover, in the karyotype of *A. cruentus*, small clusters of AmC4 were detected in the long arms of chromosome pair 7 and the pericentromeric region of chromosome pair 17. Clusters of AmC9 were revealed in the pericentromeric region of chromosome pairs 12, 15, and 16; in the terminal region of the long arms of chromosome pair 7; in the distal regions of both arms of chromosome pair 13; and in the terminal region of the short arms of chromosome pair 17 (Figure 2 and Figure 4).

In the karyotype of *A. hypochondriacus*, clusters of AmC4 were localized in the pericentromeric regions of chromosome pairs 7 and 15. Clusters of AmC9 were observed in the pericentromeric region of chromosome pair 5 and in the distal region of the short arms of chromosome pair 16 (Figure 3 and Figure 4).

## 3. Discussion

Plant genomes contain a large portion of repetitive DNA sequences [55]. Transposable elements (TEs) can constitute up to 90% of the genomes of some plants (e.g., in maize) [56]. TEs play essential roles in genome organization and evolution since they can change their copy number and location in genomes [57,58,59]. These elements are classified into two subcategories, class I (RNA transposons of retrotransposons) and class II (DNA transposons) [57,59]. LTR (Long Terminal Repeat) retrotransposons contain the Ty1-Copia and Ty3-Gypsy superfamilies, which, in turn, include a number of families mostly specific to a species or a group of related species [60]. LTR retrotransposons can contribute to variations in the nuclear genomes of plants [61,62,63,64,65]. They can replicate via copy-and-paste mechanisms and thus increase the genome size [58]. However, these retroelements can reduce the genome through both solo LTR formation and the accumulation of deletions [61]. The genome size is considered to be an intrinsic property of a plant species, and intra- and interspecific variations in genome size could be related to the different changes that occur during speciation [66]. According to different recent reports, the average genome sizes of *A. cruentus* (1C = 370.9–399 Mb) and *A. hypochondriacus* (1C = 404–466 Mb) are rather small [31,33,35]. In both species, LTR retrotransposons make up an essential proportion of their repeatomes (11% and 10%, respectively). However, plants with larger genome sizes usually contain more LTR retrotransposons compared to the studied amaranths [61,62,67]. For example, in maize with 1C= about 2.4 Gb, the proportion of LTR retrotransposons can reach up to 75% of its nuclear DNA [62]. At the same time, between the studied species, we detected interspecific variations in the genome proportions of some elements of class I (Tork, non-chromovirus Athila, chromovirus Tekay, and unclassified LTR elements) and class II (CACTA and MuDR_Mutator). Our results are mostly consistent with the repeatomic data reported earlier for other accessions of these species [35]. Moreover, the comparative repeatome analysis revealed interspecific variations in the total ribosomal DNA content, which was notably higher in *A. cruentus* compared to *A. hypochondriacus*. The observed interspecific differences might be related to processes that occur in the genomes of these *Amaranthus* species during speciation. It was previously found that some genome changes, which occurred in plants during evolution, are correlated with variations in the abundance of the SIRE (Ty1-Copia), Athila (Ty3-Gypsy), and CACTA (DNA transposon) lineages [64].

Our results showed that the genomes of the studied species contain rather small genome portions of satellite DNA sequences, which could be due to the small sizes of their genomes. The satellite DNA proportion revealed that the genome of *A. cruentus* was higher compared to *A. hypochondriacus*. Satellite DNA is considered to be a fast-evolving fraction of a plant’s repeatome, and divergences in both copy numbers and sequences were revealed even between closely related species [66]. Satellite DNA sequences have variable repeat lengths and form tandem arrays that can span up to 100 Mb [51,55]. Their genome abundance can vary even between generations, which can result in a high level of polymorphism in the length of satellite arrays [68]. The evolution of species-specific satDNA may result from copy number changes in a library of satellite sequences common to a group of species [68,69]. The sequences of some satellite DNA, however, remain unchanged for long periods of evolution [69,70], which may be due to their interaction with specific proteins required for the formation of heterochromatin, as well as their putative regulatory role in gene expression [68,71,72].

A high-throughput analysis of plant satellitomes provided important data on the structural diversity of satDNA [73,74]. In the genomes of the studied accessions of *A. cruentus* and *A. hypochondriacus*, 13 and 7 (respectively) satDNA families were identified using TAREAN pipelines. BLAST detected high sequence similarity between the most abundant satDNAs identified in genomes of both species, AmC4 (*A. cruentus*), AmH51 (*A. hypochondriacus*), and AmH9 (*A. hypochondriacus*), as well as between AmC9 (*A. cruentus*), AmC27 (*A. cruentus*), and AmH4 (*A. hypochondriacus*), confirming close relationships between these species. Moreover, sequence similarities were revealed between the identified abundant satDNAs and several RNA sequences belonging to another *Amaranthus* species (*A. tricolor*), which indicated that these species might share a common evolutionary ancestor. Our results are consistent with the earlier reported data indicating the presence of a common ancestor in species from the subgenus *Amaranthus* [36].

Moreover, our results showed that, in the *A. tricolor* genome, exon 3 of the gene, from which RNA XR_009039170.1 is transcribed, includes a tandem repeat that had 88% identity with pericentromeric repeat AmC4 (and AmH9) and covered of 38% of its length. It was previously reported that some isolated and tandem repeats are expressed as RNA transcripts in the genomes of both prokaryotes and eukaryotes [75]. The expression of pericentromeric satDNAs has been revealed in many plant species [76,77]. It has been shown that the transcription of pericentromeric satDNAs, presented in the form of small interfering RNAs, plays an important role in maintaining the structures and functioning of centromeres and in the formation of heterochromatin [78,79]. Thus, our results do not exclude the possibility of transcriptional activity in the pericentromeric satDNAs identified in the genomes of the studied species.

Among the representatives of the genus *Amaranthus*, three basic chromosomal numbers have been revealed: n = 14 (e.g., *A. tenuifolius* Willd.), n = 16 (e.g., *A. caudatus*, *A. hypochondriacus*, *A. hybridus* L., and *A. quitensis* Kunth), and n = 17 (e.g., *A. cruentus*, *A. powellii* S. Wats., and *A. palmeri* S. Wats.). In several species of the genus, variability in chromosome numbers has been revealed [42,45,80]. In the present study, 2n = 2x = 34 chromosomes were detected in the karyotypes of the studied accession of *A. cruentus*, and 2n = 2x = 32 chromosomes were found in *A. hypochondriacus*. It was previously suggested that a decrease in the basic chromosome number from n = 16 to n = 14 could result from the aneuploidy or dysploidy of their karyotypes [81,82]. An analysis of meiosis in some amaranth species, as well as interspecific hybrids, indicated that haploid number n = 17 could have evolved from n = 16 through primary trisomy [83].

Recent studies based on high-quality chromosome-level genome assemblies for *A. cruentus* and *A. hypochondriacus* using NGS technologies [33,34,35] have confirmed the close relationships between *A. cruentus*, *A. hypochondriacus*, and *A. tricolor*. Comparative genome analysis has shown that whole-genome duplication could have happened in the last ancestral species of the subgenus *Amaranthus*, and during speciation, the process of the subsequent genome diploidization of the species of this subgenus was probably accompanied by variability in basic chromosome numbers. In particular, this indicates that chromosomal loss and fusion events occurred in *A. cruentus* and *A. hypochondriacus*—followed by genome duplication—that were common to both species, as well as the fission of one chromosome in *A. cruentus*, which resulted in a haploid chromosome number of 17 in its karyotype [35].

The rDNA families; the 18S, 5.8S, and 26S rRNA genes (45S rDNA); and the 5S rRNA genes are essential constituents of eukaryotic genomes, which are involved in the processes of protein synthesis [84,85,86]. The sequences of 45S rDNA and 5S are often used as chromosome markers in FISH-based comparative karyological analyses due to their relatively conserved nature and abundance as ‘house-keeping genes’ [86]. Moreover, they are widely used in phylogenetic studies of plant species [87]. In the present study, we observed one pair of chromosomes bearing large clusters of 45S rDNA in karyotypes of both *A. cruentus* and *A. hypochondriacus*. The FISH results are consistent with previously published data demonstrating one pair of chromosomes bearing 45S rDNA signals in both species [45,46]. At the same time, we detected three (*A. cruentus*) and four (*A. hypochondriacus*) chromosome pairs with 5S rDNA signals, confirming the presence of interspecific diversity between genomes of *A. cruentus* and *A. hypochondriacus* [45]. In *A. cruentus*, intraspecific variability in the chromosome distribution patterns of 5S rDNA loci (in 3–4 chromosome pairs) has also been reported [45]. These results show that 5S rDNA is involved in the process of speciation within *Amaranthus*.

SatDNAs are often associated with heterochromatin regions and localized in certain chromosome regions (centromeric, terminal, and/or intercalary), which allow them to be used in FISH assays for comparative karyotype analyses [52,53,54]. The patterns of chromosomal distribution in satDNAs facilitate the recognition of homologous chromosome pairs, as well as differences between lineages and species [53,87]. In the present study, the high sequence homology of certain satDNAs identified in the genomes made it possible to use AmC9, AmC4, and AmC12 as oligonucleotide FISH probes for the comparative karyotype analyses of *A. cruentus* and *A. hypochondriacus*. At the same time, the localization of satDNAs in chromosomes can be clustered and/or dispersed, which is probably related to the different amounts and organizations of the repeats in the genomes of the studied taxa [47,69,70]. In our study, one of the abundant repeats, AmC12, presented dispersed localization in chromosomes of both species, and it was excluded from further karyotype analyses. Both AmC4 and AmC9 demonstrated unique clustered species-specific patterns in their distribution in the chromosomes of both *A. cruentus* and *A. hypochondriacus*, which made it possible to identify chromosome pairs in the karyotypes. This indicates that AmC4 and AmC9 could be used as new, promising chromosomal markers for comparative karyotype studies within the genus Amaranthus.

In the karyotypes of both species, significant similarity in the chromosome morphology and patterns of chromosome distribution in the studied molecular markers was observed in eleven chromosome pairs, which could confirm their close relationships, shown earlier by the chromosome-level genome assembly approach [35]. In the other five chromosome pairs, the analysis of the chromosome distribution patterns of the studied markers indicated the possible chromosome rearrangements that could occur in these species during speciation. Finally, in *A. cruentus*, one middle-sized metacentric chromosome pair (14) did not have any putative homologous pair in *A. hypochondriacus* karyotype, and this chromosome probably belongs only to the *A. cruentus* chromosome set. At the same time, the analysis of collinearity between the pseudo-chromosomes of *A. cruentus* and *A. hypochondriacus* [35] indicated a fission of one chromosome in *A. cruentus* (after copy loss and fusion in other chromosomes shared with *A. hypochondriacus*) to produce n = 17. Consistent with this, Ma et al. suggested that this chromosome (after the fission) should be the shortest among the 17 chromosomes of *A. cruentus* [35]. These findings indicate the need for further studies of the karyotypes of various accessions of both species.

## 4. Materials and Methods

### 4.1. Plant Material

Seeds of *Amaranthus cruentus* (Pk-318, Russia) and *A. hypochondriacus* (Vk-811, Russia) were obtained from the seed collection of the FRC N.I. Vavilov All-Russian Institute of Plant Genetic Resources (VIR).

### 4.2. Chromosome Spread Preparation

The seeds were germinated for 3–4 days at room temperature (RT) in Petri dishes on moist filter paper. Root tips (0.5–1 cm long) were excised and treated with a saturated aqueous solution of α-monobromonaphthalene (10–20 mL of α-monobromonaphthalene should be mixed with 3×volume of distilled water and shaken vigorously) at RT for 3–4 h to accumulate metaphases. Then, the root tips were placed in ethanol/glacial acetic acid (3:1) fixative at 4 °C for 48 h. The fixed roots were transferred into 1% acetocarmine solution in 45% acetic acid for 20 min. Then, each root was placed on the slide and squashed using a cover slip. After freezing in liquid nitrogen, the slide was dehydrated in 96% ethanol and air-dried.

### 4.3. Sequence Analysis and Identification of DNA Repeats

The genome sequences of both *A. cruentus* and *A. hypochondriacus* were used for the genome-wide analyses and the identification and characterization of major repeat families using RepeatExplorer and TAREAN pipelines [49,50]. For *A. cruentus*, 25,000,000 paired-end reads (150 bp in length) were selected from the basecalled sequencing data of a publicly available sample (accession number SRX10357816; https://www.ncbi.nlm.nih.gov/sra/SRX10357816 accessed on 12 March 2021). For *A. hypochondriacus*, 36,000,000 paired-end reads (100 bp in length) were used from the basecalled paired-end genome sequences of a publicly available sample (accession number ERR3021343; https://www.ncbi.nlm.nih.gov/sra/ERR3021343 accessed on 14 January 2020). Both amaranth samples were sequenced with Illumina HiSeq 4000. This granted at least 10× the coverage for the *A. cruentus* genome and 8× the coverage for the *A. hypochondriacus* genome with respective genome sizes of 1C = 370.9 Mb and 1C = 466 Mb [31,35]. The genomic reads were filtered by quality, and 1,321,949 (*A. cruentus*) and 2,503,965 (*A. hypochondriacus*) high-quality reads were randomly selected for further analyses, which corresponded to approximately 0.5× the coverage of the genomes of both *A. cruentus* and *A. hypochondriacus*, which was within the limits recommended by the developers of these pipelines (genome coverage of 0.01–0.50× is recommended) [50].

The RepeatExplorer2/TAREAN software was launched with preset settings based on the Galaxy platform (https://repeatexplorer-elixir.cerit-sc.cz/galaxy; accessed on 17 February 2024). Each repeat proportion was calculated using RepeatExplorer as the ratio of the number of reads specific to a particular repeat type to the sum of all reads used in the cluster analysis. The sequence homology of the satDNAs identified in the genome of *A. cruentus* with repeats, which were revealed earlier in other taxa, was estimated using BLAST (NCBI, Bethesda, MD, USA). Based on three most abundant satDNAs of *A. cruentus*, which exhibited high sequence homology with DNA repeats of *A. hypocondriacus*, oligonucleotide FISH probes, AmC4, AmC9, and AmC12 (Table 3), were generated using the Primer3-Plus software (https://www.primer3plus.com/index.html, accessed on 16 December 2024) [88].

### 4.4. Multicolor Fluorescence in Situ Hybridization

For sequential MC-FISH assays, we used a combination of five labeled DNA probes. Two wheat DNA probes—pTa71, containing 18S-5.8S-26S (45S) rDNA of common wheat [89], and pTa794, containing 5S rDNA of common wheat [90]—were labeled directly with fluorochromes Aqua 431 dUTP or Red 580 dUTP (ENZO Life Sciences, New York, NY, USA) using the Nick Translation DNA Labeling System 2.0 (ENZO Life Sciences, New York, NY, USA). Additionally, we used three oligonucleotide DNA probes, AmC4, AmC9, and AmC12 (Table 1), which were synthesized with the labeled nucleotides ROX-dUTP and 6-FAM-dUTP in *Syntol* (Moscow, Russia).

FISH assays were carried out according to a previously described protocol with minor modifications [87]. Chromosome slides were pretreated with RNAse A (Roche Diagnostics, Mannheim, Germany) dissolved in 2 × SSC (1 mg/mL) for 1 h at 37 °C. After the pretreatment, the slides were washed in 2 × SSC at RT three times for 10 min each; dehydrated through a graded ethanol series (70%, 85%, and 96%) for 2 min each; and air dried. Then, 40 ng of each labeled probe was dissolved in the hybridization mixture (50% formamide and 70% hybridization specificity (stringency) in a total volume of 15 μL) and dropped onto each slide. Then, the slide was covered with a coverslip, sealed with rubber cement, denatured at 74 °C for 5 min, chilled on ice, and placed in a moisture chamber at 37 °C for 16–20 h. The slides were washed in 0.1 × SSC for 5 min at 42 °C and then in 2 × SSC for 5 min at 42 °C (80% of stringency of the post-hybridization washes), followed by a 5 min wash in PBS at RT. The slides were dehydrated, air-dried, and stained with DAPI (4′,6-diamidino-2-phenylindole) dissolved (0.1 μg/mL) in Vectashield mounting medium (Vector Laboratories, Burlingame, CA, USA). After processing the FISH results, the chromosome slides were washed in distilled water for 5 min. Then, a sequential FISH procedure was conducted on the same slides.

### 4.5. Chromosome Analysis

Chromosome slides were analyzed using the epifluorescence Olympus BX61 microscope with a standard narrow band pass filter set and a UPlanSApo 100/1.40 oil UIS2 objective (Olympus, Tokyo, Japan). Chromosome images were captured at the same magnification in grayscale channels with a monochrome CCD (charge-coupled device camera) (Cool Snap, Roper Scientific, Inc., Tucson, AZ, USA). The obtained images were pseudo-colored and processed using Adobe Photoshop 10.0 (Adobe, San Jose, CA, USA) and the VideoTesT-FISH 2.1 (IstaVideoTesT, St. Petersburg, Russia) software.

At least 5 plants (15 metaphase plates for each plant) were analyzed. Chromosome pairs in karyotypes were identified according to the chromosome size, morphology, and distribution patterns of the studied markers. The chromosome pairs in the karyograms were set in a decreasing order of size.

## 5. Conclusions

Our results demonstrate that cytogenomic studies of *Amaranthus* species can provide valuable data on the genomic relationships between species. The FISH-based chromosome distribution patterns of the combination of four molecular markers (45S rDNA, 5S rDNA, AmC4, and AmC9) revealed the similarity of the karyotypes of *A. cruentus* and *A. hypochondriacus,* which indicates their common ancestry. The interspecific differences in the patterns of the chromosome distribution of 5S rDNA, AmC4, and AmC9 loci, detected between the studied species, could be related to genome changes that occurred during speciation. Two new satDNA-based chromosomal markers, AmC4 and AmC9, are particularly useful for the identification of amaranth chromosomes with small sizes. They could be useful for comparative cytogenomic investigations within the genus *Amaranthus* to increase knowledge on genome organization in these valuable crops.

## Figures and Tables

**Table 1:** Proportions of major DNA repeats identified in genomes of *Amaranthus cruentus* and *Amaranthus hypochondriacus* using RepeatExplorer.

Repeat Name	Genome Proportion, %	
	*A. cruentus*	*A. hypochondriacus*
Retrotransposons (Class I)	11.06	10.07
Ty1 Copia	4.28	4.00
Ale	0.29	0.29
Angela	0.12	0.13
Bianca	0.12	0.11
Ikeros	0.05	-
Ivana	0.06	0.06
SIRE	1.43	1.62
TAR	0.86	0.86
Tork	1.35	0.93
Ty3-Gypsy	5.37	3.06
Non-chromovirus Athila	1.44	1.07
Non-chromovirus Tat-Retand	0.53	0.41
Chromovirus CRM	0.49	0.28
Chromovirus Tekay	2.90	1.30
Chromovirus Reina	0.01	-
LINE	0.54	0.66
Unclassified LTR elements	0.87	2.35
Transposons (Class II)	3.52	3.07
CACTA	0.82	1.24
MuDR_Mutator	1.46	0.77
hAT	0.53	0.39
PIF_Harbinger	0.06	0.05
Tc1_Mariner	0.42	0.38
Helitron	0.23	0.24
Ribosomal DNA	5.06	3.54
Unclassified repeats	11.5	15.21
DNA satellite	0.27	0.16
Putative satDNA families	6 high confident	7 low confident
	3 high confident	4 low confident

**Table 2:** Comparison of sequences of the most abundant satDNAs identified by TAREAN in genomes of *Amaranthus cruentus* and *Amaranthus hypochondriacus*.

Tandem Repeat/Cluster Proportion, % *A. cruentus A. hypochondriacus*		Repeat Length, bp	BLAST Similarity
AmC4/1.0 (88% identity with AmH51 99% identity with AmH9)	AmH51/0.45 AmH9/0.14 (85% identity with AmH51)	169 *	AmC4 88% identity/38% cover with *A. tricolor* uncharacterized LOC130799021, ncRNA, Sequence ID: XR_009039170.1 Gene ID: 130799021, Exon 3
AmC9/0.55 AmC27/0.23 (86% identity with AmC9)	AmH4/1.5 (100% identity with AmC9)	42 *	AmC9 95% identity/9% cover with *A. tricolor* uncharacterized LOC130813864, transcript variant X2, ncRNA, Sequence ID: XR_009042247.1 Gene ID: 130813864, Exon 7
AmC12/0.47	AmH26/0.24 (97% identity/ 41% cover with AmC12)	3008 (AmC12) 3949 (AmH26)	AmC12 92% identity/100% cover with *A. tricolor* uncharacterized LOC130817693, mRNA, Sequence ID: XM_057683543.1 Gene ID: 130817693, Exon 1, 2; Intron 1

* The repeats have the same length unless otherwise stated.

**Table 3:** List of generated oligonucleotide FISH probes.

Tandem Repeat/Genome Proportion [%]	Sequences of the Generated Oligonucleotide FISH Probes
CL4/1.0	AmC4 ACACTATTTGGTATATATTATTGTGTTGAAGTAGTTAGAATCGAAAATAATTGTCATATGCTTGAAATTAAGTGTTAAGTTGCGTTTTTAAGGGTTTTGAACTATTTTTGTCACTTTCGCGCGTAAAATAGCTTAAACTTGGTTTGTTATGCACGAAACTTGGCACACA
CL9/0.55	AmC9 CATTGTTCATTGATCATTGATCCTTGTTCATTGTTCATCGTT
CL12/0.47	AmC12_1 TTTTGAAGTTGAGTGTGATGCATCTGGGGTAGGTATTGGAGGTGTCCTAACTCAAAACA ACAAACCTCTTGCTTATTTT AmC12_1196 ACGTGTGCATATAGTTTGGTTATTGTTCGACACGTAGCCAACCTATATCATCTTGGTATCAGAGCCAAGGCTACGCTCC AmC12_2443 GGCAAGGTATGTTCTCTTATTATTGATGGAGGAAGTTGCACTAATGTTGCTTCAAAGACTATGGTGGACAAGCTT

## Data Availability

All data generated or analyzed during this study are contained within the article and Supplementary Materials.

## Supplementary Materials

The following supporting information can be downloaded at https://www.mdpi.com/article/10.3390/ijms252413575/s1.
