# Supplementary material for: Genome Studies in Amaranthus cruentus L. and A. hypochondriacus L. Based on Repeatomic and Cytogenetic Data
Source: Int J Mol Sci. 2024 Dec 18;25(24):13575. doi: 10.3390/ijms252413575 (PMC11678860; doi:10.3390/ijms252413575)
Supplement: Supplementary file 1 [file ijms-25-13575-s001.zip › Supplementary Figures.pdf]

Supplementary Figures

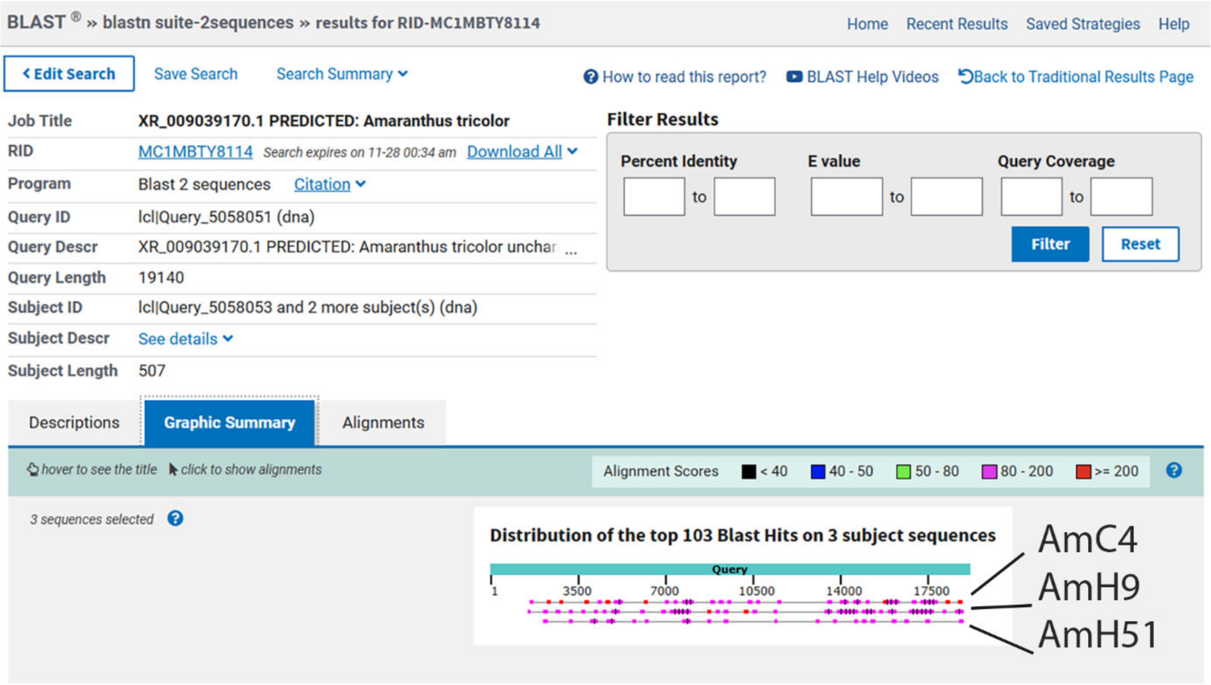

Supplementary Figure S1. BLAST results showing the extent of alignment of satDNAs AmC4, AmH9, and AmH51 on the external sequence *Amaranthus tricolor* uncharacterized LOC130799021, ncRNA, Sequence ID: XR_009039170.1

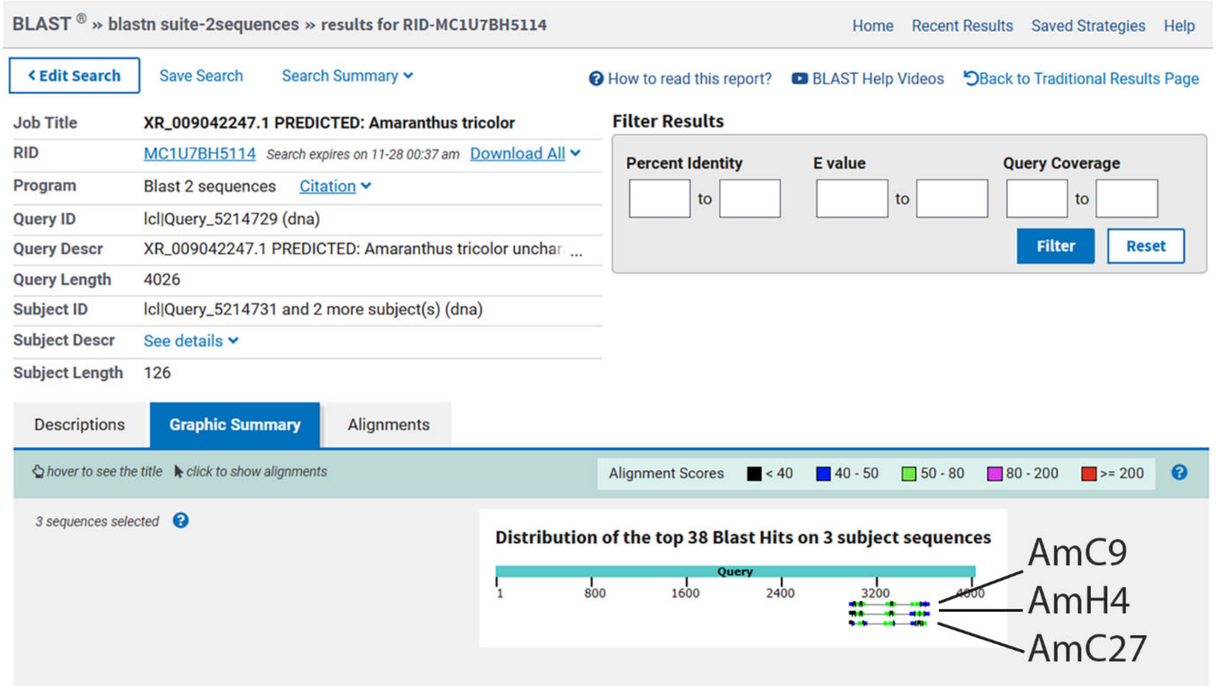

Supplementary Figure S2. BLAST results showing the extent of alignment of satDNAs AmC9, AmH4, and AmC27 on the external sequence *Amaranthus tricolor* uncharacterized LOC130813864, transcript variant X2, ncRNA, Sequence ID: XR_009042247.1.

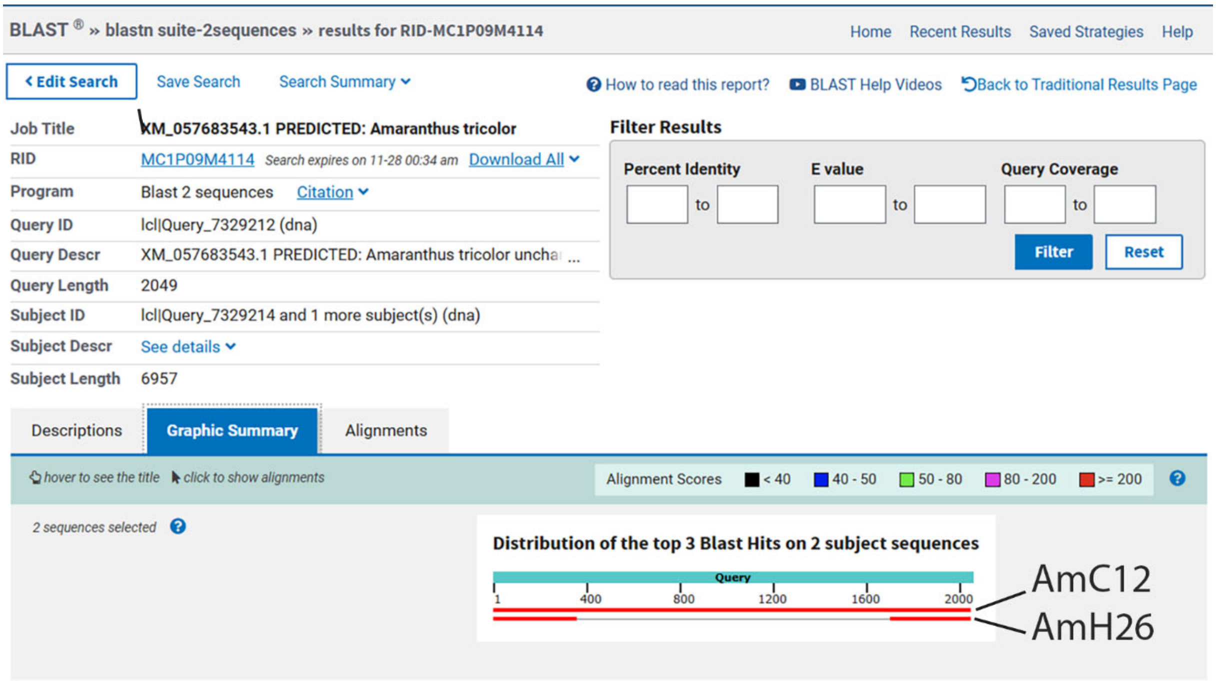

Supplementary Figure S3. BLAST results showing the extent of alignment of satDNAs AmC12 and AmH26 on the external sequence *Amaranthus tricolor* uncharacterized LOC130817693, mRNA, Sequence ID: XM_057683543.1

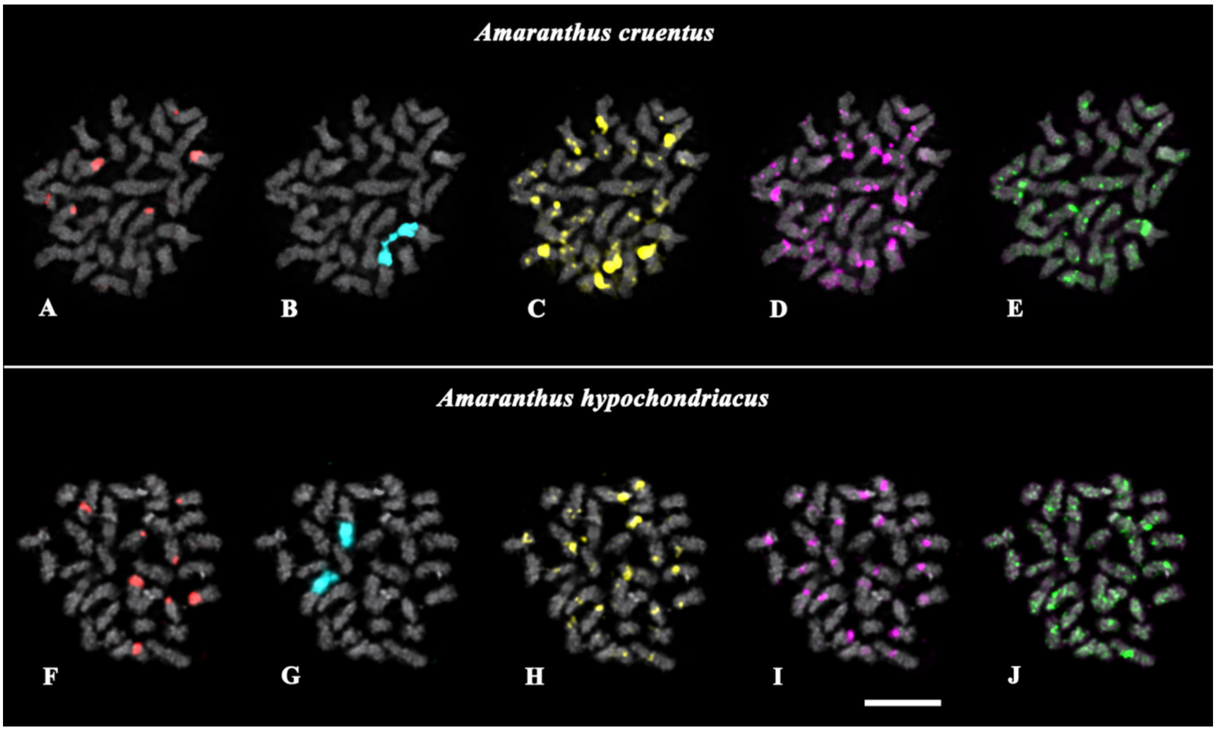

Supplementary Figure S4. FISH-based mapping of (A, F) 5S rDNA (red); (B, G) 45S rDNA (aqua); (C, H) AmC4 (yellow); (D, I) AmC9 (purple); and (E, J) AmC12 (green) in karyotypes of *Amaranthus cruentus and Amaranthus hypochondriacus*. DAPI-staining – dark grey. Scale bar – 5 µm.
